# Exploring the therapeutic potential of different sources of mesenchymal stem cells: a novel approach to combat burn wound infections

**DOI:** 10.3389/fmicb.2024.1495011

**Published:** 2024-11-29

**Authors:** Shahrzad Aliniay-Sharafshadehi, Mohammad Hasan Yousefi, Mohammad Ghodratie, Mojtaba Kashfi, Hamed Afkhami, Seyed Mehdi Ghoreyshiamiri

**Affiliations:** ^1^Department of Microbiology, Faculty of Advanced Science and Technology, Tehran Medical Science, Islamic Azad University, Tehran, Iran; ^2^Department of Tissue Engineering and Applied Cell Sciences, School of Medicine, Qom University of Medical Sciences, Qom, Iran; ^3^Cellular and Molecular Research Center, Qom University of Medical Sciences, Qom, Iran; ^4^Department of Medical Microbiology, Faculty of Medicine, Bushehr University of Medical Sciences, Bushehr, Iran; ^5^Fellowship in Clinical Laboratory Sciences, Mashhad University of Medical Sciences, Mashhad, Iran; ^6^Nervous System Stem Cells Research Center, Semnan University of Medical Sciences, Semnan, Iran; ^7^Department of Medical Microbiology, Faculty of Medicine, Shahed University, Tehran, Iran; ^8^Tehran University of Medical Sciences, Tehran, Iran

**Keywords:** mesenchymal stem cells (MSCs), burn injury, infection, stem cell-based therapy, wound healing

## Abstract

The most prevalent and harmful injuries are burns, which are still a major global health problem. Burn injuries can cause issues because they boost the inflammatory and metabolic response, which can cause organ malfunction and systemic failure. On the other hand, a burn wound infection creates an environment that is conducive to the growth of bacteria and might put the patient at risk for sepsis. In addition, scarring is unavoidable, and this results in patients having functional and cosmetic issues. Wound healing is an amazing phenomenon with a complex mechanism that deals with different types of cells and biomolecules. Cell therapy using stem cells is one of the most challenging treatment methods that accelerates the healing of burn wounds. Since 2000, the use of mesenchymal stem cells (MSCs) in regenerative medicine and wound healing has increased. They can be extracted from various tissues, such as bone marrow, fat, the umbilical cord, and the amniotic membrane. According to studies, stem cell therapy for burn wounds increases angiogenesis, has anti-inflammatory properties, slows the progression of fibrosis, and has an excellent ability to differentiate and regenerate damaged tissue. Figuring out the main preclinical and clinical problems that stop people from using MSCs and then suggesting the right ways to improve therapy could help show the benefits of MSCs and move stem cell-based therapy forward. This review’s objective was to assess mesenchymal stem cell therapy’s contribution to the promotion of burn wound healing.

## Graphical abstract

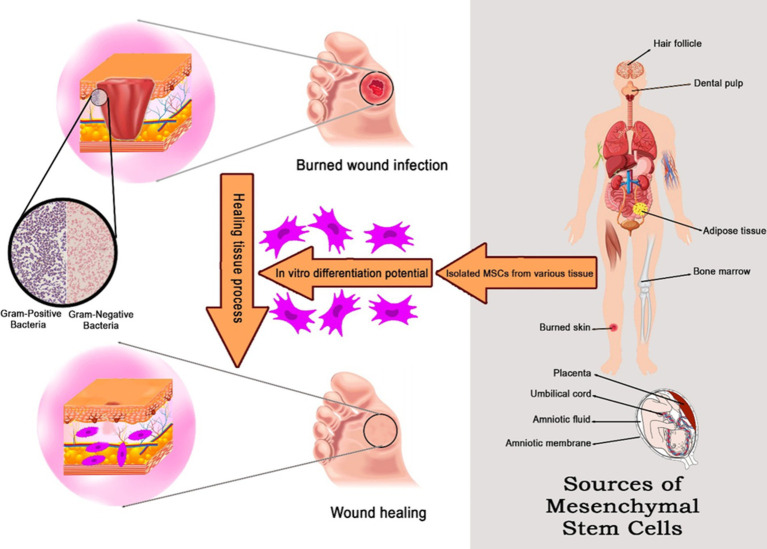

## 1 Introduction

One of the worst and most excruciating injuries anybody may experience is a burn injury (Kumari and Nanda, 2022). Burns are frequently understood to be skin sores caused by exposure to high temperature or heat, electricity, chemicals, or radiation (Jahromi et al., 2018). Thermal injuries account for 5–20% of all injuries and 4% of all fatalities (Schulman et al., 2022). Over 265,000 people worldwide die from burns each year (Wang et al., 2023). Burn injuries and the likelihood of dying from those injuries are both affected by age, occupation, and socio-economic status. Older buildings, lax safety standards, a lack of smoke alarms, and faulty electricity all lead to a higher risk of burn death and injury in low-development countries (Opriessnig et al., 2023).

The most common cause of mortality following a burn injury is wound infection (Wang et al., 2018). The body’s primary line of protection against dangerous foreign microbes is the skin. Burning destroys the skin’s integrity, allowing bacteria to enter and cause illness (Sun et al., 2022). In addition, burn infections hinder wound healing (Xiong Y. et al., 2023). Patients with severe burns are more prone to infection because their cutaneous barrier has been compromised and their systemic immune responses have been changed. The most prevalent cause of death in individuals with severe burns is septicemia, which occurs when bacteria infiltrate the deeper layers of damaged tissue and travel into the bloodstream (Sarker et al., 2022). Common pathogenic bacteria found in infected burn patients include *Staphylococcus aureus*, *Pseudomonas aeruginosa*, *Acinetobacter baumannii*, *Klebsiella pneumoniae*, and other coliform bacteria. Antimicrobial resistance is a major obstacle to treating many bacterial infections. Many studies have identified the most prevalent multidrug-resistant (MDR) bacteria in burn units (El Hamzaoui et al., 2020).

Despite general advancements in the treatment of individuals with acute burn injuries, morbidities related to more severe burn injuries continue to be widespread. Too frequently, burn victims experience severe tissue loss, scarring, and contractions that impair physical function and have long-term psychological and emotional effects (Schulman et al., 2022). In addition, large areas of deep burn wounds will disrupt the internal milieu and induce both local and systemic organ dysfunction if they are not treated quickly and effectively (Zhang et al., 2023). Over the past 10 years, significant progress has been made in the treatment and study of burn injuries. Examples of these developments include the creation of novel skin substitutes, the use of novel antimicrobial wound dressings and improved systemic drug delivery for the treatment of wound infection, the testing of novel pharmacological interventions, the identification of new targets for the control of wound pain, and sophisticated surgical techniques such as laser therapy, fat grafting, skin grafting, and coverage options such as design of a hydrogel system (Wang et al., 2018; Li et al., 2024).

Since stem cells have a higher capacity for regeneration and support the healing and regeneration processes in multiple ways, they offer numerous advantages over typical treatments based on growth factors or cytokine biologicals. Specifically, mesenchymal stem cells (MSCs) have demonstrated conclusive therapeutic benefits on a variety of tissue damage (Hu et al., 2022; Afkhami et al., 2023). MSCs are able to differentiate into many cell types and also have a robust ability for cell proliferation. They have the ability to differentiate into different types of tissues, such as bone, cartilage, adipose tissue, tendons, and muscles. The use of MSCs to speed the healing process after skin injuries, such as burns, has increased dramatically in recent years (Wang et al., 2021; Andalib et al., 2023).

One of the earliest studies on the use of MSCs to treat burn wounds in rats was reported in 2003 by Shumakov et al.; they utilized embryonic lung fibroblasts and bone marrow-derived MSCs in this research. According to their findings, MSCs have the ability to speed up the healing process of wounds and even rebuild damaged skin tissue (Gardien et al., 2014). Fu et al. (2004) treated minipigs with deep partial-thickness burns with MSCs and basic fibroblast growth factor (bFGF) (Rodgers and Jadhav, 2018). In 2005, MSCs were initially used to heal burns in people in Russia. Promising results were observed in five female patients treated with allogeneic MSCs (Mahmoudian-Sani et al., 2018). It is important for researchers to carefully consider the advantages and disadvantages of each MSC source. Patients with severe burns, for instance, may not be able to extract bone marrow. In contrast, a less invasive method can obtain MSCs in large quantities from adipose tissue (Ozturk and Karagoz, 2015).

The aim of this review is to investigate the treatment of burn wounds using stem cells derived from bone marrow, fat, umbilical cord, amniotic membrane, amniotic fluid, placental tissue, hair follicles, and dental pulp.

## 2 Skin structure and classification of burns

Regarding the application of MSCs in burn wounds, it is essential to comprehend normal skin structure and the pathological mechanisms of the skin following a burn (Xiong W. et al., 2023). The skin is the largest organ in the human body. In adults, it makes up approximately 15% of body weight and has an area of 1.5 to 2 m^2^. Skin is a vital organ that performs a variety of biological tasks, including excretion, heat management, vitamin D synthesis initiation, protection from toxins and infections, and hydration. Therefore, severe skin injuries may be dangerous (Tottoli et al., 2020; Huynh et al., 2022).

The skin is divided from top to bottom into three layers: the epidermis, dermis, and hypodermis (Jahromi et al., 2018; Andalib et al., 2023). There are five layers that make up the epidermis, arranged from outermost to lowest: the stratum corneum (SC), stratum lucidum, stratum granulosum (granular layer), stratum spinosum (spinous layer), and stratum basale (basal layer). From the basal layer, keratinocytes eventually separate and move outward. The viable epidermis is made up of the final four layers. Keratinocytes mature into corneocytes when they reach complete maturity. The dermis layer, which is made up of connective tissue and contains the skin’s hair follicles, sebaceous glands, and sweat glands, is located underneath the epidermis layer (Rahma and Lane, 2022).

**Figure 1:**
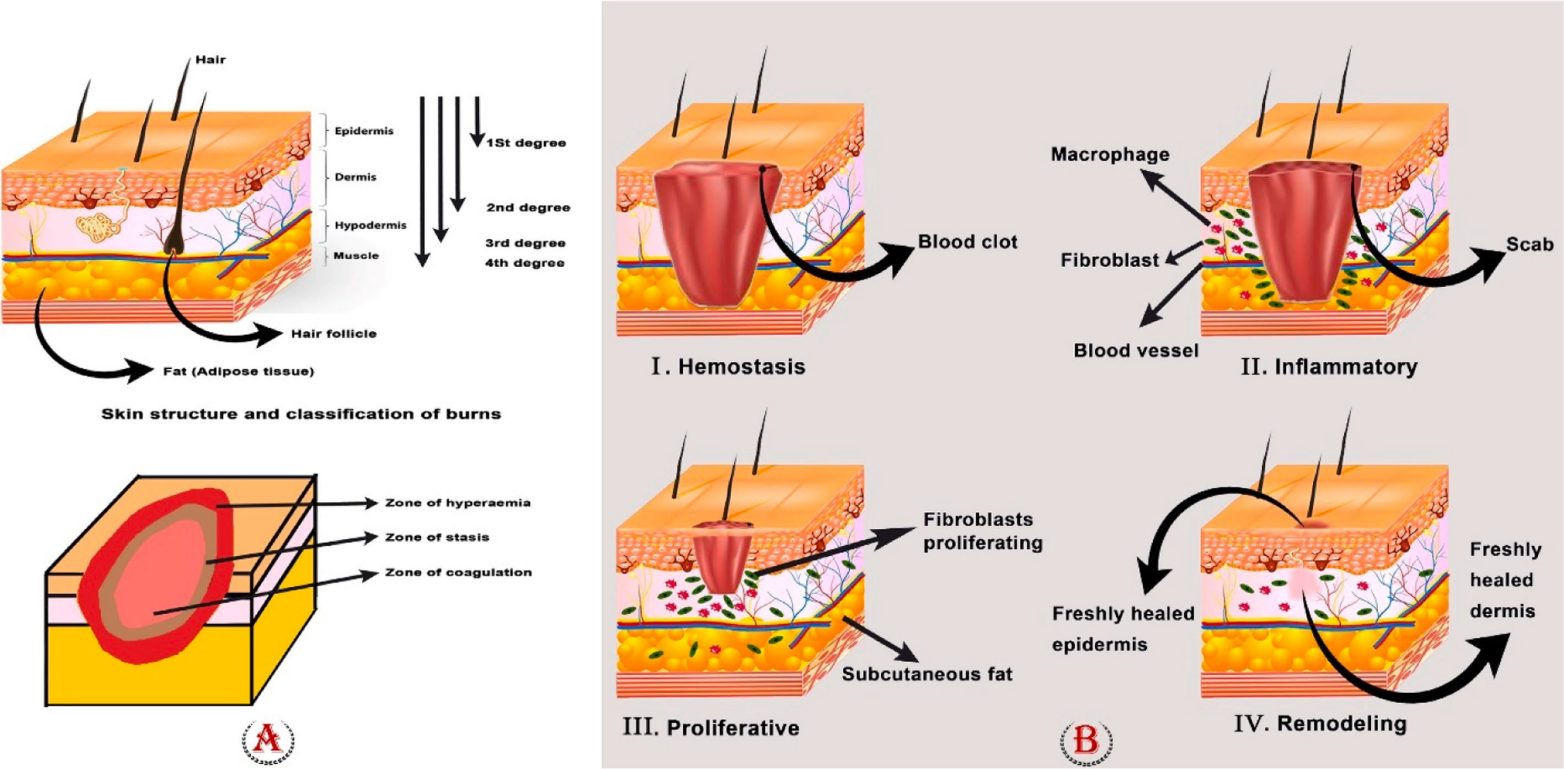
**(A)** Skin structure, classification of burns, and zones of the burn area. To determine the appropriate care for a patient, it is crucial to identify the depth of the burn. First-degree burns (those that involve only the outer layer of skin) are completely harmless, although they can be excruciatingly painful. They heal without leaving scars and typically do not require surgery. If a burn reaches the dermis, it is considered a second-degree or partial-thickness burn, and it will likely cause painful blisters. When third-degree (full thickness) or fourth-degree burns show, surgical intervention is required. **(B)** Four phases of wound healing: hemostasis, inflammation, proliferation, and remodeling phase.

The epidermis controls body temperature, the dermis preserves structural integrity and aids in feeling, and the hypodermis serves as mechanical protection and regulates body temperature (Irfan et al., 2022). Skin injuries are classified depending on the depth of their penetration into the skin, the amount of tissue destruction, and the percentage of total body surface area (TBSA) of the damaged wound (Xiong W. et al., 2023; Irfan et al., 2022) (Figure 1).

First-degree burns, deep or superficial second-degree burns, third-degree burns, and fourth-degree burns are the different categories of burn injuries. Burns of the first degree only affect the epidermis, which is the skin’s outermost and superficial layer. In this instance, the epidermis turns red, and the pain lasts only a short while. Surface burns of the second degree cause destruction to the whole epidermis and a portion of the nipple layer. The skin turns red, swells, and hurts; it needs wound care to heal and typically recovers more quickly than deep second-degree wounds. The papillary dermis is injured in second-degree severe burns, although the dermis and skin appendages are still there. Edema is visible, and the skin turns red and white. Surgery is required for this kind of burn, and there are scars. In third-degree burns, the epidermis, dermis, underlying skin tissues, and even muscles and bones are destroyed. The wound surface is covered with necrotic tissue. Third-degree burns require surgery and will leave a large scar. Burns of the fourth degree injure the underlying muscle or bone more deeply. Due to the elimination of nerve endings, third- and fourth-degree burns are not painful. They require surgery and meticulous burn care to avoid infection. Loss of the affected area is often common in fourth-degree burns (Kumari and Nanda, 2022; Xiong W. et al., 2023; Luck et al., 2021).

Furthermore, burns can be categorized as major or minor. A minor burn is one that covers less than 10% of the TBSA. The definition of a major burn is not always clear. Guidelines for categorizing serious burn injuries are as follows: more than 20% TBSA in adults, more than 30% in children, and more than 10% in elderly patients (Jeschke et al., 2020).

## 3 Zones of the burn area

The tissue damage model proposed by Jackson categorizes tissue injury into three distinct zones, namely, the zone of coagulation, the zone of stasis, and the zone of hyperemia. Necrosis, loss of plasma membrane integrity, and denaturation of constituent proteins can all be seen in the coagulation zone. When it is possible, the tissue in the zone of coagulation is surgically removed because necrotic tissue is prone to infection and slows healing. Necrosis can be avoided by carefully controlling the stasis zone to treat ischemia. Yet abrupt tissue reperfusion may cause the release of inflammatory cytokines, which could lead to reperfusion damage. Vasodilatation caused by local inflammatory mediators and viable cells creates a zone of hyperemia. Unless there is a serious infection or hypoperfusion, the tissues in this zone eventually heal fully (Johnson et al., 2022; Abazari et al., 2022) (Figure 1).

## 4 Healing of burn wounds

Burn wound healing is a complex and vital mechanism that involves a number of molecular and cellular processes (Kumari and Nanda, 2022). Slow and incomplete healing can cause serious harm, including the loss of hair, glands, skin, and, in severe situations, tissue death (Sasmal and Ganguly, 2023). Four interrelated and overlapping phases make up this process: hemostasis, inflammation, proliferation, and remodeling (Abazari et al., 2022). Different growth factors, cytokines, chemokines, and various cells all play a role in coordinating the overlapping phases of wound healing (Su et al., 2019).

In brief, hemostasis is triggered to stop blood loss as soon as damage is sustained. The inflammatory response begins to eradicate the invading pathogens and prepare the tissue for healing. During the proliferative phase, neovascularization, fibroblast migration, and re-epithelialization occur in a suitable way. Ultimately, in the remodeling stage, a scar takes the place of the granulation tissue (Ma et al., 2023; Fakouri et al., 2024) (Figure 1).

### 4.1 Hemostasis

Usually, burn wounds do not bleed right away after being injured. Still, hemostasis and vasoconstriction are the first steps in the healing process for burn wounds, just like they are for other traumas (Johnson et al., 2022). Constriction of the damaged vessels initiates hemostasis quickly after injury, preventing excessive blood loss. This process, also known as the pro-inflammatory stage, starts as soon as the damage occurs. After tissue damage, prostaglandin 2-*α* and thromboxane A2 are released into the injured area, leading to an extreme vasoconstrictor reaction that lasts for 5 to 10 min. The small vessels in the wound are then constricted to achieve hemostasis. Vasodilation, or the enlargement of blood vessels, comes next and reaches its peak 20 min after it. As a result, tissue hypoxia and acidosis develop, dampening the vasoconstrictive effect and heightening vascular permeability for inflammatory cells. At this point, platelets are crucial because they initiate the clotting process and release numerous signaling molecules such as platelet-derived growth factor (PDGF), epidermal growth factor (EGF), fibronectin, fibrinogen, histamine, serotonin, and the Von Willebrand factor (Abazari et al., 2022; Lasocka et al., 2019).

### 4.2 Inflammation

The inflammatory phase attracts more cells to the wound site, including neutrophils, mast cells, monocytes, and T lymphocytes. When a wound is this far along in its healing process, it shows changes in a number of different substances, including transforming growth factor-*β* (TGF-β), tumor necrosis factor alpha (TNF-α), EGF, PDGF, VEGF, FGF, IL-1, IL-6, IL-8, and IL-12. These mediators influence angiogenesis, collagen synthesis, epithelialization, and the regulation of the inflammatory process (Lasocka et al., 2019).

Macrophages stimulate cell growth and migration by secreting FGF, PDGF, VEGF, and TGF-α/TGF-*β*. In addition, macrophages eliminate pathogens and debris from the injury site (Bai et al., 2022). During the early phases of healing, M1 macrophages play a role in phagocytic activity, phagocytosing neutrophils, and eliminating any remaining bacteria or debris in a wound. The transition from M1 to M2 initiates fibroblast proliferation and angiogenic activity by producing anti-inflammatory mediators and extracellular matrix (ECM) (Miricescu et al., 2021). Other than leukocytes, regulatory T cells (Tregs) can control tissue inflammation by reducing the production of interferon-*γ* (IFN-γ) and augmenting the number of macrophages that promote inflammatory responses (Sorg et al., 2017). Once macrophages begin secreting EGF and PDGF, they create granular tissue, allowing tissue regeneration to progress into the proliferative phase (Abazari et al., 2022).

### 4.3 Proliferation

This stage involves the repair of blood vessels and the formation of granulation tissue at the wound site through a series of continuous processes, including angiogenesis, fibroblast migration, and epithelialization. It begins 4 days after the injury and lasts for 2 weeks (Abazari et al., 2022; Ayavoo et al., 2021; Fakouri et al., 2024). Angiogenesis occurs under particular conditions, such as low oxygen tension and low pH, in the wound’s area. Fibroblasts, macrophages, and epidermal cells produce bFGF, VEGF, and TGF-*β*, which promote it. Fibroblasts are the most common cells during the proliferative phase because they are the ones that create the new matrix needed to restore the structure and form of injured tissue. TGF-β and PDGF cause fibroblast proliferations, which are released from a hemostatic clot and migrate to the damaged arteries (Ayavoo et al., 2021). At this time, activated fibroblasts that have differentiated into myofibroblasts begin contracting the wound’s edges (Guillamat-Prats, 2021).

Fibrin, fibronectin, and collagen are all products of fibroblast cells attaching to a matrix. Tissue granulation begins at the wound site when collagen and fibronectin are secreted. Finally, epithelial cells build a full layer on the wound by increasing their mitotic activity, and they are attached to the cell matrix. The final step is the creation of the basement membrane by the aggregation of mature epithelial cells (Ayavoo et al., 2021). When the wound is fully covered and an intact epithelial barrier is reestablished, the process of epithelial migration and proliferation ends. M2 macrophages control the proliferation and migration of keratinocytes, fibroblasts, and endothelial cells, which leads to tissue regeneration and an abundance of ECM. High amounts of immature collagen type III are secreted into the matrix by fibroblasts (An et al., 2021).

### 4.4 Re-modeling

In the final stage of wound healing, termed as remodeling or maturation, cellular and vascular components gradually decrease. This phase normally begins after the finalization of granulation tissue growth and lasts for a very long time, anywhere from 21 days to a year following the injury (Qin et al., 2023). Wound re-epithelization by keratinocytes and ECM deposition by fibroblasts and endothelial cells mark the beginning of the remodeling phase of wound healing (El Ayadi et al., 2020). The last stage of wound healing ends when scar formation occurs (Sorg et al., 2017), which is largely dependent on how the collagen fibers are arranged. Small, parallel bundles of collagen are characteristic of normal scars, while hypertrophic scars are characterized by thinner, more numerous, and more cross-linked collagen fibers (El Ayadi et al., 2020). In the following, collagen I begins to gradually replace collagen III (Kirby et al., 2015). Collagen I is deposited more slowly than collagen III but has greater tensile strength and thus replaces collagen III in the ECM (El Ayadi et al., 2020). Matrix metalloproteinases (MMPs) and tissue inhibitors of metalloproteinases (TIMPs) are the main factors that regulate this process (Kirby et al., 2015).

## 5 Burn wound infections

Burn wound infection (BWI) has always been a great challenge of burn care (Guo et al., 2022). After extensive burn damage, multiple infectious problems may arise because the skin barrier has been damaged and the host’s cellular and humoral immune responses have been dampened both locally and systemically (Vinaik et al., 2019). The microorganisms that colonize burn wounds come from the endogenous flora of the patient. On the other hand, polluted hospital surfaces, water, pollutants, air, and healthcare personnel’s hands could also transmit them to the patient (Al-Maliki et al., 2022).

Microorganisms are present in nearly every wound; however, not all wounds become infected (Ding et al., 2022). Scar tissue is made up of avascular necrotic tissue and forms on the surface of deep partial-thickness and full-thickness burns. The scar provides a location rich in favorable proteins for microbial colonization and growth. Scar tissue not only limits host immune cell movement but also blocks the delivery of systemic antimicrobials to the site of injury. Also, the release of toxic compounds from the scar disrupts the majority of the host’s immunological responses. In addition to the nature and severity of thermal injury, the kinds and number of microorganisms present at the burn site influence the wound infection (Kirby et al., 2015).

According to research, wound infections account for 42 to 65% of burn-related fatalities (Maitz et al., 2023). Infection is caused by a high concentration of bacteria (>10^5^ CFU) in and around the wound (Msheik et al., 2023). Burn wound infection usually occurs in the acute phase after injury. Patients of different ages experience significant changes in the incidence of infection. In general, compared to other age groups, elderly people (over 55 years old) and young children (under 4 years old) are more likely to get wound infections and mortality (Zhang et al., 2021). A person’s immune system, the depth of the wound, and the surrounding environment all play a role in what kinds of microorganisms infect the skin and the wound. Generally, wound infections caused by bacteria, fungi, or viruses on the skin can occur (Table 1) (Solanki and Nagori, 2013).

**Table 1:** Common microorganisms responsible for burn wound infections.

Group	Species	References
Gram-positive bacteria	*Staphylococcus aureus* *Streptococcus pyogenes* Methicillin-resistant *S. aureus* Coagulase-negative staphylococci *Enterococcus* spp. (including Vancomycin resistant Enterococci)	Maitz et al. (2023) and Vathulya et al. (2022)
Gram-negative bacteria	*Pseudomonas aeruginosa* *Acinetobacter baumannii* *Stenotrophomonas maltophilia* *Vibrio* spp. *Aeromonas* spp. *Chryseobacterium Indologenes* *C. memingosepticum* *Burkholderia cepacia Escherichia coli* *Klebsiella pneumonia* *Enterobacter* spp. *Citrobacter* *Serratia marcescens* *Proteus* spp.	Maitz et al. (2023) and Vathulya et al. (2022)
Anaerobic bacteria	*Bacteroides* spp. *Peptococcus* spp. *Clostridium* spp. *Fusobacterium* spp. *Actinomyces* spp. *Peptostreptococcus* spp. *Finegoldia* spp. *Prevotella* spp. *Porphyromonas* spp.	Maitz et al. (2023) and Andalib et al. (2023)
Fungi	*Candida* spp. *Aspergillus* spp. *Fusarium* spp. *Alternaria* spp. *Rhizopus* spp. *Mucor* spp.	Maitz et al. (2023) and Vathulya et al. (2022)
Viruses	Herpes simplex virus Cytomegalovirus Varicella-zoster virus Human papilloma virus	Maitz et al. (2023) and Vathulya et al. (2022)

One major challenge is the prevalence of organisms that are resistant to drugs. Methicillin-resistant *S. aureus* (MRSA) is the most common resistant bacterium in burns, typically treated with the antiquated antibiotic vancomycin (Branski et al., 2009; Diederen et al., 2015). The resistance of organisms to antibiotics has led to the repurposing of old antibiotics. Recently, researchers have reinvigorated the antibiotic colistin, which caused side effects such as neurotoxicity and nephrotoxicity, to combat MDR. Researchers have demonstrated favorable therapeutic properties by conjugating colistin to dextrin in nano-antibiotic therapeutic polymers (Azzopardi et al., 2013a,b).

## 6 Mesenchymal stem cells

Alexander Fridenstein first defined MSCs in the 1960s. MSCs are adult multipotent stromal progenitor cells that are heterogeneous, non-hematopoietic, and capable of self-renewal as well as differentiation into many lineages and cell types (Al-Anazi et al., 2020; Mahjoor et al., 2023a). There is some evidence that stem cell origin, proliferation procedures, and the culture microenvironment all have a role in MSCs’ ability to differentiate (Yu et al., 2023). MSCs have been widely used in the fields of tissue engineering and regenerative medicine due to their low immunogenicity, high capacity for self-renewal, multidirectional differentiation, and comparatively simple, non-invasive access.

MSCs are almost always found in all tissues and have certain similarities in their phenotype, structure, and functions. Therefore, several sources have been proposed for their isolation (Najar et al., 2022). Bone marrow, adipose tissue, and the umbilical cord have been the main sources of MSCs for therapeutic use (Álvarez-Viejo, 2020). MSCs should have the following properties, as per the guidelines laid out by the International Society of Cell Therapy (ISCT) Committee: I. proficiency in adhering to and growing on plastic in a controlled laboratory setting; II. the capacity to undergo *in vitro* cell differentiation into osteoblasts, adipocytes, and chondroblasts; III. the presence of MSC-specific markers (Dabrowska et al., 2021).

MSCs lack the expression of markers such as CD45, CD35, CD19, CD11b, CD34, CD14, CD79α, and human leukocyte antigen-DR (HLA-DR) while expressing CD105, CD73, CD71, CD44, CD271, and CD90. Because MSCs lack MHCII and have low MHC1, they are immunologically inactive. Tissue regeneration is made possible by MSCs because of their immunomodulatory properties and their ability to transdifferentiate into different cell types. MSCs release prostaglandins, chemokines, and cytokines that impact the function of immune cells. In addition, they increase the production of regulatory T cells and subtypes of anti-inflammatory macrophages (Abdul Kareem et al., 2021; Zawrzykraj et al., 2023; Bhujel et al., 2023; de Witte et al., 2016). After MSCs are grafted into the host, they have been shown to be very immunogenic (Yu et al., 2023).

Various clinical investigations have demonstrated the usefulness of both autologous and allogeneic MSCs as sources for tissue formation. Specifically, researchers have assessed the safety of administering autologous MSCs and their capacity to reduce immunological risk. Research by Falanga et al. has shown that using autologous bone marrow mesenchymal stem cells (*BM*-*MSCs*) to treat wounds is an effective and safe option (Margiana et al., 2022).

### 6.1 BM-MSCs

BM-MSCs are a class of heterogeneous cells made up of multipotent stem cells that Frieden first recognized (Gao et al., 2022). Just 0.002% of all stromal cells are BM-MSCs (Mazini et al., 2019). They have the capacity to differentiate into a variety of cell types, including osteoblasts, chondrocytes, myocytes, adipocytes, epithelial cells, neuron cells, fibroblasts, myofibroblasts, keratinocytes, and endothelial cells (Revilla et al., 2023). The first type of cells to be employed in burn wound therapy is BM-MSCs. It has been demonstrated that BM-MSCs have the capacity to promote angiogenesis, scarless healing, and enhanced collagen formation, all of which are crucial factors in effective wound healing.

BM-MSCs have several benefits, but low yield and restricted availability of donors are among their disadvantages (Abdul Kareem et al., 2021). Despite the traditional harvesting of stem cells from the bone marrow, which is an invasive method with low efficiency, the ability of cell fusion enables them to regenerate tissue and immunity (Francis et al., 2019). BM-MSCs have the ability to modulate the immune system. They directly inhibit the proliferation of monocytes, DCs, and inflammatory T cells. In addition, they produce mediators that reduce inflammation, including IL-1Ra, PGE2, IDO, and IL-10 (Gherghel et al., 2023) (Table 2).

**Table 2:** Comparison of types of MSCs.

Cell types	Advantages	Disadvantages	Immunological response and wound healing mechanisms	References
BM-MSCs	Rapid cell proliferation and differentiation Differentiation capability multidirectional Long-term differentiation capabilities	Limited access to donors Harvesting cells invasively and painfully Limited self-renewal ability A significant decrease in the number, differentiation potential, and lifespan of these cells with increasing age Easily infected	Prevent monocyte, DC, and inflammatory T-cell proliferation Produce anti-inflammatory IL-1Ra, PGE2, IDO, and IL-10	Abdul Kareem et al. (2021), Mazini et al. (2019), Francis et al. (2019), Gherghel et al. (2023), Cheng et al. (2018), Luan et al. (2021), and Hassanshahi et al. (2019)
AD-MSCs	Ease of access Availability of abundant amounts Less invasive source High yield Few morbidity among donors during collection Immunosuppressive High cellular activity Release of growth factors	Limited self-renewal ability Impacted by donor age Longer duplication period in comparison with BM-MSCs. Lower osteogenic and chondrogenic potential than BM-MSCs.	Diminish inflammation by upregulating chemokines and cytokines via Th cells and IL-10 Healing by paracrine activity.	Gherghel et al. (2023), Aghayan et al. (2022), Luan et al. (2021), Hassanshahi et al. (2019), Dini et al. (2022), and Berebichez-Fridman and Montero-Olvera (2018)
UC-MSCs	High proliferation capacity Contains anti-inflammatory qualities Harvesting cells non-invasively Fewer ethical issues Lower risk of infection Minimal teratoma formation Low immunogenicity with immunosuppressive properties	Limited access Low survival rate *in vivo* The adipogenic capacity of UC-MSCs is debatable. Its osteogenic potential is lower than BM.	By inhibiting inflammatory cell infiltration, lowering IL-6, 1, and TNF-a, and increasing IL-10 and TSG-6, UC-MSCs accelerated wound healing. Presence of the TNF-stimulated gene/protein 6 (TSG-6) anti-inflammatory mechanism.	Xiong W. et al. (2023), Lukomskyj et al. (2022), Phan et al. (2023), Nourian Dehkordi et al. (2019), Luan et al. (2021), Berebichez-Fridman and Montero-Olvera (2018), and Hwang et al. (2023)
PL-MSCs	Capability to attain maximal cell count Ability to harvest utilizing non-invasive methods Maintaining high proliferation capacity for a minimum of twenty passages Less immunological response Influence immunomodulatory	Lower migration speed compared to BM-MSCs Less differentiated to adipogenic lineage than BM-MSCs	PL-MSCs have high levels of PD-L1 and PD-L2, which may limit T-cell proliferation by pausing the cell cycle.	Abd-Allah et al. (2015), Makhoul et al. (2013), Shojaei et al. (2019), Luan et al. (2021), and Díaz-Prado et al. (2011)
AM-MSCs	Low cost source Low toxicity Minimal immunogenicity Antimicrobial impact Immunomodulatory effect	Donor screening Threat of disease transmission Difficult suturing Differences in biological characteristics based on factors such as gestational age, sample location, donor age, and race	AM-MSCs are the sources of keratinocyte growth factors (KGFs) and EGF. AECs produce chemicals that inhibit T and B lymphocyte proliferation and neutrophil and macrophage chemotactic capacities. AM attaches to T cells and other leukocytes, reducing inflammation. The AM supports neovascularization and wound repair by absorbing and transplanting endogenous progenitor cells. AM releases chemokines and cytokines that modulate the wound immunological response, reducing local inflammation.	Aghayan et al. (2022), Bernabé-García et al. (2021), and Fairbairn et al. (2014)
AF-MSCs	High proliferative potential Easy harvesting Self-renewal ability No ethical issues Minimal immunogenicity Multipotent stem cells with broad distinction Higher impact on immunomodulation compared to BM-MSCs	Harvesting in the second or third trimester might cause infection, early delivery, and injury to the infant or mother.	High quantities of α-defensin, lysozyme, calprotectin, cathelicidin, TGF*α*, TGFβ1, IGF1, and EPO are found in AF.	Nejad et al. (2021), Harrell et al. (2019), Mankuzhy et al. (2021), Luan et al. (2021), Min-hong (2020), and Subhan et al. (2021)
HF-MSCs	Ease of access An abundance of sources Robust cell proliferation Broad differentiation potentials Low immunogenicity lack of age limits No ethical issues Non-carcinogenic	No problems were reported	HF-MSCs can differentiate into keratinocytes, inter-follicular epidermis, sweat glands, sebaceous glands, and HFs. As a result, it may promote wound healing. HF-MSC promotes the transition from fibroblasts to myofibroblasts in wounds *in vivo*, shortening the proliferation stage.	Abdul Kareem et al. (2021), Liu et al. (2024), Li S. et al. (2022), and Zaki et al. (2020)
DP-MSCs	High content of cells Low invasive procedures More angiogenesis than BMSCs and AD-MSCs in burn wound healing	Difficult preparation Periodontal and ectomesenchymal tissues impact MSC characteristics.	The DP-MSCS may stimulate endogenous stem cells to repair injured tissues by paracrine effect, which releases angiogenic factors, cytokines, chemokines, and exosomes DP-MSCs express CD73 highly.	Abbas et al. (2018), Dini et al. (2022), and Berebichez-Fridman and Montero-Olvera (2018)
BD-MSCs	Simple and inexpensive cell harvesting Non-invasive procedure No ethical issues Low immune response and rejection	Effect of heat degradation on cell types in severe burns Their migration into burnt tissue may take time.	BD-MSCs promote healing because they exhibit the myofibroblast phenotype, which is responsible for producing ECM and contracting wounds.	Surowiecka et al. (2022), Amini-Nik et al. (2018), and Van Der Veen et al. (2012)

In a research by Xue et al., BM-MSC was injected into 30 mice that had burn injuries. In with the control group, the recovery period shortened from 25 to 20 days (Purwanthi, n.d.). Singer et al. conducted a similar investigation using a rat model and found that injecting rat BM-MSCs intravenously can postpone the course of burn damage in a rat comb-burn model measured from the necrotic region (Singer et al., 2013). Formigli et al. (2015) used a mouse model to study what happened when BM-MSCs were transplanted onto bioengineered scaffolds that had platelet-rich plasma (PRP) in them. The test results showed evidence of improved skin regeneration quality, less collagen deposition, more neoangiogenesis, and repaired sebaceous glands and hair follicles (Formigli et al., 2015).

Aryan et al. (2019) conducted a study with the aim of determining whether hBM-MSC can enhance wound healing in deep second-degree burns in male rats. They randomly divided 32 adult male rats per time point into four groups: (1) control group, (2) sham group (DMEM), (3) common treatment group (CT), and (4) conditioned media group (CM). They reported that, relative to the control and DMEM groups, the CM and CT groups demonstrated a significant enhancement in wound closure on the 15th and 28th days after the burn injury. In addition, hBM-MSC facilitated increased cell proliferation, and it promoted both collagen synthesis and angiogenesis at the injury site (Aryan et al., 2019).

### 6.2 Adipose-derived MSCs

The first study to describe AD-MSCs was by Zuk in 2001, which piqued the interest of researchers (Czerwiec et al., 2023). AD-MSCs exist between adipocytes and the vascular endothelium. Adipose tissue can be used to easily separate 100% of local MSCs, and retrieval does not need cell culture. These stem cells, which can repair all layers of skin, can also be extracted from discarded burn skin (Abdul Kareem et al., 2021). Fat stem cell research on mice has demonstrated that by boosting tissue renewal and cell proliferation, it also boosts the development of new blood vessels and the control of proteins (Lukomskyj et al., 2022). Furthermore, the advantages of AD-MSCs are their high availability, minimum invasiveness, and no restrictions (Nourian Dehkordi et al., 2019). Zhu et al. (2012) demonstrated that AD-MSCs grew and doubled quicker than BM-MSCs (Abu-El-Rub et al., 2024). Compared to BM-MSCs, adipose tissue extraction has fewer risks and negative effects. They can also develop into multiple cell lineages and produce nutrients and immune-modulating substances (Goncharova et al., 2019).

The best option in regenerative medicine is AD-MSCs since they are readily available, contain macrophages, fibroblasts, and endothelial stem cells, and have incredible pluripotent potential (Abdul Kareem et al., 2021). AD-MSCs reduce the inflammatory response by upregulating chemokines and cytokines through Th cells and IL-10 (Liu et al., 2018). Zhang et al. (2018) found that AD-MSCs’ paracrine activity boosts wound healing (Jo et al., 2021). Researchers have conducted clinical studies with AD-MSCs using both autologous and allogeneic transplants. The allograft method’s low immunological rejection risk is another benefit of AD-MSCs, but there are a few drawbacks to take into account before using AD-MSCs. This specific cell population has limited ability to self-renew (Czerwiec et al., 2023).

AD-MSCs are widely used in plastic surgery, and depending on the type of adipose tissue that is harvested, they have different functions. White adipose tissue, which is extracted from visceral and subcutaneous surfaces, has the ability to store triglycerides, and brown adipose tissue is extracted from the neck, mediastinum, supraclavicular, and par scapular regions. They are involved in the production of body heat, the ability to modulate the immune system, neoangiogenesis, and endogenous repair; they cause wound regeneration; and by releasing hormones such as leptin, they can be effective in wound healing (Francis et al., 2019). Paganelli et al. (2019) used MSCs sourced from adipose tissue to construct a dermal replacement for wound healing. This substitute exhibited excellent mechanical qualities and remarkable biocompatibility (Jo et al., 2021) (Table 2).

### 6.3 Umbilical-derived MSCs

One of the finest sources of MSCs is the umbilical cord. They include both hematopoietic and non-hematopoietic cells, as well as endothelial progenitor cells. Different stem cell types may be extracted from the numerous layers of the umbilical cord, including Wharton’s jelly, veins, arteries, the lining of the cord, and the sub meningeal and perivascular areas (Abdul Kareem et al., 2021; Phan et al., 2023). Due to their adaptability, simplicity in isolation and culture, rapid proliferation, easier differentiation, and immunosuppressive qualities, umbilical cord-derived MSCs have enormous potential as a therapeutic tool in tissue engineering and regenerative medicine (Jo et al., 2021; Phan et al., 2023).

According to the studies, it was shown that the umbilical cord stem cell has a high ability to heal burn wounds by increasing skin appendages and creating fibers. However, this source has more limited access compared to fat tissue and bone marrow (Lukomskyj et al., 2022). Umbilical cord cells have abilities such as detecting inflammatory tissues, differentiating to prevent inflammation, and performing anti-inflammatory activities. By releasing cytokines, they cause wound tissue repair and regeneration. In laboratory conditions, they have the ability to transfer to the body and differentiate into efficient cells (Atluri et al., 2020). Studies in rats indicated that UC-MSCs hastened wound healing by blocking inflammatory cell infiltration, decreasing IL-6, 1, and TNF-a, and boosting IL-10 and TSG-6 (Nourian Dehkordi et al., 2019). According to a study by Liu et al. (2016), UC-MSCs lessened acute inflammation in rats that had suffered severe burns. They demonstrated the TNF-stimulated gene/protein 6 (TSG-6) anti-inflammatory mechanism (Xiong W. et al., 2023).

An abundant supply of MSCs can be found in the umbilical cord’s Wharton’s jelly-like matrix (Nazempour et al., 2020). MSCs from Wharton’s jelly (WJ-MSCs) promote wound healing by reducing inflammation (Mansour et al., 2023). There are several advantages to using WJ-MSCs, including its accessibility to a broad pool of donors, ease of acquisition, lack of danger to the donor, lack of ethical constraints, low immunogenic potential, and high differentiation capability (Owczarczyk-Saczonek et al., 2018) (Table 2). They show significant immunomodulatory activity similar to BM-MSCs (Nazempour et al., 2020). These cells have the potential to transform into cells that gland-like cells, which speeds up the healing process of the skin (Hashemi et al., 2020). In addition, the donor is completely safe because contact with infectious pathogens is so infrequent (Owczarczyk-Saczonek et al., 2018).

### 6.4 Placenta-derived MSCs

Over a century has passed since placental tissue (PL) was first used to treat wounds (Azzopardi et al., 2013b). PL is an excellent source of growth factors and stem cells, which are vital to healing and creating new tissues (Teoh et al., 2023). There are several benefits associated with the placenta: the ability to obtain the maximum number of cells; the possibility of harvesting them using non-invasive techniques; a lower immune response than BM-MSCs due to their embryonic origin; and favorable immunomodulatory effects *in vitro* (Abd-Allah et al., 2015; Maxson et al., 2012) (Table 2).

In 2006, Chang et al. showed that PL-MSCs had a much stronger immunosuppressive impact than BM-MSCs (Malek and Bersinger, 2011). PL-MSCs differentiate into adipocytes, osteocytes, chondrocytes, endothelial cells, and neuronal cells (Maxson et al., 2012; Makhoul et al., 2013). Compared to BM-MSCs, PL-MSCs exhibit greater proliferation ability. However, Li et al. showed that BM-MSCs migrate faster than PL-MSCs, which suggests that BM-MSCs are better able to get through the endothelial blood vessel barrier (Makhoul et al., 2013). PL-MSCs’ ability to stimulate neovascularization, wound diminution, and enhanced blood flow speeds up the healing process (Shojaei et al., 2019).

Research has shown that PL-MSCs exhibit high quantities of the cell adhesion molecules programmed death ligands 1 and 2 (PD-L1 and PD-L2), which may block T-cell proliferation by stopping the cell cycle (Shojaei et al., 2019).

### 6.5 Amniotic membrane-derived MSCs

The amniotic membrane (AM) has been the subject of extensive studies as a cell source for regenerative therapies. This membrane is responsible for the fetus’s physical defense, pH control, and the release of anti-inflammatory substances (Miatmoko et al., 2023). The AM lacks vascular tissue and contains a three-layer structure. It consists of an epithelial cell monolayer, an acellular intermediate layer, and an outer layer that contains MSCs (Roubelakis et al., 2012). Generally, the AM includes amniotic membrane-derived MSCs (AMSCs), amniotic epithelial cells (AECs), and fibroblasts (Hu et al., 2023; Nejad et al., 2021). The AM-MSCs have the ability to differentiate into cells of the ectodermal, mesodermal, and endodermal layers (Kim et al., 2014).

The epithelium seems to be a source of biologically significant stem cells. Extracellular matrix proteins such as collagen and fibronectin make up the basement membrane. Both the stroma and the spongy layer contain vital chemicals and agents for regeneration (Pfister et al., 2023). AECs secrete several substances that suppress T and B lymphocyte proliferation as well as the chemotactic abilities of neutrophils and macrophages in inflammatory circumstances such as wounds. Another interesting finding is that AM binds to T cells and other leukocytes, preventing them from taking part in the inflammatory process (Aghayan et al., 2022).

AM-MSCs are the sources of keratinocyte growth factors (KGF) and EGF (Miatmoko et al., 2023). Since 1910, the usage of human amniotic membrane (hAM) grafts in the therapy of burn wound healing has increased significantly (Rahman et al., 2019). The AM influences wound healing through the absorption and transplantation of endogenous progenitor cells, which promotes neovascularization and wound repair. AM decreases the local inflammatory response as a result of the release of chemokines and cytokines that regulate the immune response at the site of the wound. Consequently, AM can have a favorable impact on wound healing (Pfister et al., 2023).

Studies using mouse models have demonstrated that AM usage decreases local inflammation, promotes cell renewal, and also promotes collagen formation. In addition, the use of AM was linked to a reduction in the number of bacteria in infected mouse burn wounds. In a sheep model, Fraser et al. demonstrated that AM therapy at burn sites considerably reduced the quantity of scar tissue (Pfister et al., 2023). Some advantages of using amniotic membrane stem cells in the healing process include alleviating pain, reducing inflammation, regulating fluid loss, decreasing bacterial colonization, preventing scars, the minimum ethical considerations, and low immunogenicity. All of these advantages make amniotic membrane stem cells appealing to people and give them the chance to be used in cell therapy and regenerative medicine (Hu et al., 2023; Pfister et al., 2023) (Table 2).

### 6.6 Amniotic fluid-derived MSCs

Amniotic fluid (AF) is a feeding and protecting liquid that helps the embryo develop normally (Harrell et al., 2019). Amniotic fluid (AF) contains high concentrations of *α*-defensin, lysozyme, calprotectin, and cathelicidin, as well as TGFα, TGFβ1, insulin-like growth factor 1 (IGF1), and erythropoietin (EPO) (Nyman et al., 2022). AF-MSCs were first identified as a source for wound healing in 2013 (Rahman et al., 2019). AF-MSCs are obtained from AF samples during the second trimester (16–28 weeks) through amniocentesis (Harrell et al., 2019; Anudeep et al., 2022). Many research studies have shown that volume, donor variability, and gestational stage are the three most important factors that affect the number of cells obtained from amniocentesis (Teoh et al., 2023) (Table 2).

The risks of AF harvesting during the second or third trimester include harm to the mother or fetus, infection, premature labor, and even miscarriage. Nonetheless, AF obtained following cesarean section births appears to be a non-invasive, abundant, and high-yield cell source for cell treatment (Mankuzhy et al., 2021). AF-MSCs are adult, fibroblast-like, self-renewable, multipotent stem cells with broad distinction and minimal immunogenicity (Harrell et al., 2019). AF-MSCs express a variety of antigens, such as HLA-ABC, CD73, CD44, CD105, CD166, CD117, CD29, CD49e, CD58, and CD90, but these cells do not express hematopoietic markers such as CD34, CD14, CD45, CD133, CD31, and HLA-DR (Mankuzhy et al., 2021).

### 6.7 Hair follicle-derived MSCs

The hair follicle (HF) is a dynamic little organ that supports several vital bodily biological processes. HFs are a readily available source of stem cells that may self-renew, differentiate, control hair growth, and help maintain skin homeostasis. Research has demonstrated that hair follicle stem cells (HFSC) are both multipotent and extremely proliferative in laboratory settings (Mistriotis and Andreadis, 2013). HFSCs exhibit immunological rejection, making them the ideal donors for cell-based therapies. According to Li et al.’s study, HFSCs are superior to other cell types at repairing wounds (Abdul Kareem et al., 2021).

Studies on the usage of HF-MSCs in wound healing are still limited (Ou et al., 2020). There are a number of benefits to using HF-MSCs, including an abundance of sources, ease of access, robust cell proliferation, broad differentiation potentials, low immunogenicity, lack of age limits, no ethical issues, and non-carcinogenic (Liu et al., 2024; Li S. et al., 2022; Zaki et al., 2020). They have the potential to develop into HFs, sweat glands, sebaceous glands, keratinocytes, and inter-follicular epidermis. So, it has the potential to improve the healing process of wounds (Zaki et al., 2020) (Table 2).

According to reports by Kevin et al., HF-MSCs can aid in the healing of chronic wounds (Liu et al., 2024). When HF-MSC is used to treat wounds *in vivo*, it accelerates the transition from fibroblasts to myofibroblasts, which in turn reduces the duration of the proliferation stage (Yang H. et al., 2023).

### 6.8 Dental pulp-derived MSCs

Because dental pulp has a high content of cells and requires relatively few invasive methods for cell separation, it has recently been regarded as a potential source of MSCs (Abbas et al., 2018). Improvements have been shown after transplantation of DP-MSCs (derived from the teeth of adult patients), which are attributed to the production of paracrine substances by these cells (Gomes et al., 2010). Through paracrine impact, the DP-MSCS may drive endogenous stem cells to regenerate damaged tissues by secreting growth factors, cytokines, chemokines, angiogenic factors, and exosomes (Ichi et al., 2023). Studies have shown that DP-MSCs have more angiogenic potential in comparison with ASCs and BMSCs. In addition, DP-MSCs have a high level of CD73 expression (Abbas et al., 2018) (Table 2).

### 6.9 Burn-derived MSCs

Research has shown that the use of human MSCs extracted from burned skin accelerates the healing process in rat and pig burn models (Surowiecka et al., 2022). Amini-Nik et al. demonstrated for the first time the presence of viable mesenchymal skin stem cells in full-thickness burned skin. Furthermore, based on their reports, these cells were easily removed, expanded *in vitro*, and then added to wound covering in a simple and inexpensive way. The majority of patients are willing to donate these discarded tissues. Cell isolation from burned skin is a non-invasive procedure that poses no danger to the patient. Because they are the patient’s own skin stem cells, the risk of immunological response and rejection is minimal (Amini-Nik et al., 2018) (Table 2).

In another study, MSCs were extracted from the eschar by Van der Veen et al. They believed that MSCs from the burn eschar had moved from another source of MSCs, such as subcutaneous fat, into the wound region. In addition, it has been found that burn patients’ blood has higher concentrations of circulating MSCs. The function of these cells and when they are introduced to the wound site remain uncertain. These cells have the myofibroblast phenotype, which produces ECM and contracts wounds; therefore, they may aid healing (Van Der Veen et al., 2012).

## 7 Function of MSCs in burn wound healing and skin regeneration

MSCs can help with wound healing by changing into other cell types such as fibroblasts, epithelial cells, and keratinocytes. MSCs have the potential to modulate the local reparative responses in injured areas by attracting host cells, including fibroblasts, keratinocytes, macrophages, and progenitor cells. Then, the paracrine effects that lead to an increase in angiogenesis, neovascularization, de-epithelization, collagen production, and, in the end, the release of several growth factors and cytokines promote wound healing (Mahmoudian-Sani et al., 2018; Maranda et al., 2017; Mirshekar et al., 2023).

Paracrine factors increase homeostatic and anti-apoptotic genes and decrease nucleic acid, protein metabolism, and apoptotic genes (Tamama and Kerpedjieva, 2012). Following severe burn injury, the hypermetabolic response is triggered by the systemic inflammatory response, which initiates protein catabolism and breakdown. This leads to the uncontrollable release of pro-inflammatory mediators, which exacerbates organ dysfunction and protein loss (El-Sayed et al., 2024).

Van Badiavas’s laboratory has shown *in vitro* that MSCs may also promote wound healing by the production of exosomes that include transcription factors, mRNA, and miRNA that are essential for wound healing (Maranda et al., 2017). Aryan et al. (2019) used stereological techniques to examine mice with profound second-degree burns that had received human bone marrow mesenchymal stem cell-conditioned media (hBM-MSC-CM). According to this study, hBM-MSC-CM promotes basal cell and fibroblast growth, stimulates collagen and blood vessel production, and has anti-inflammatory properties that aid in the healing of skin lesions. In addition, the stereological data showed that the hBM-MSC-CM group had epithelialization with thick dermis, fibrous, and granular tissue, while the control group had less collagen production and more inflammatory cells (Aryan et al., 2019).

### 7.1 Effects of MSCs in homeostasis phase regulation

Significant amounts of phosphatidylserine and tissue factor (TF) are present on the surface of MSCs and MSC-derived extracellular vesicles (EVs), which induce coagulation. In fact, clot formation can be exacerbated because of the expression of these two elements that trigger a thrombotic reaction. Some research has demonstrated that Annexin V is on the surface of MSCs. It indicates that phosphatidylserine is present, which is what makes clots form (Guillamat-Prats, 2021).

### 7.2 Effects of MSCs on the inflammatory response

MSCs begin to have immunosuppressive effects once they arrive at injury sites (Hu et al., 2018). MSCs immediately reduce the inflammatory response. In addition, MSCs regulate it by lowering the quantity of neutrophils, macrophages, and activated T cells (Maxson et al., 2012; Mahjoor et al., 2023b). Pro-inflammatory cytokines, including IL-1, TNF-*α*, and IFN-*γ*, are reduced in MSC-treated wounds, while anti-inflammatory cytokines such as IL-10 and IL-4 are increased. The immunosuppressive phenotype of MSCs is activated with exposure to pro-inflammatory cytokines such as IFN-γ, TNF-α, IL-1α, and IL-1β, leading to the expression of chemokines and inducible nitric oxide synthase (iNOS), all of which inhibit T-cell responsiveness to inflammation (Maxson et al., 2012; Hu et al., 2018).

The presence of TNF-α and IFN-γ may increase the production of cytokines such as cyclooxygenase 2 (COX2), hepatocyte growth factor (HGF), and prostaglandin E2 (PGE2) for inhibiting T-cell proliferation. Furthermore, they aid in the synthesis of chemokines such as CCR5, CCR10, and CXCL9, which prevent the growth of immunological effector cells (Huang et al., 2022). T cells emit less IFN-*γ* and more IL-4 to respond to MSC activity. Thus, the number of regulatory T cells rises. In addition, MSCs control the growth, development, and activity of B cells and natural killer cells (NK cells), leading to reduced IFN-γ secretion by NK cells (Maxson et al., 2012; Hu et al., 2018). MSCs can cause a switch from the pro-inflammatory M1 state to the anti-inflammatory M2 state of macrophage polarization (Ulivi et al., 2014).

### 7.3 Improving the proliferative stage using MSCs

Fibroblasts and myofibroblasts are crucial during the third phase of wound healing. Proliferation and restoration of epithelial cells, as well as the synthesis of collagen and ECM proteins, occur during this phase (Andalib et al., 2023). A vast array of growth factors, including PDGF, VEGF, FGF, and tumor necrosis factor-induced Dutch gene-6 (TSG-6), are also produced by MSCs and can promote the reparative abilities of fibroblasts, endothelial cells, and tissue precursor cells. MSCs, by acting on PDGF-BB, promote the migration, secretion, and proliferation of fibroblasts (Liu et al., 2022).

### 7.4 Modifying the remodeling phase with MSCs

In the latter phases of wound healing, MSCs effectively manage matrix remodeling and scar reduction (Magne et al., 2018). MSCs play a role by secreting MMPs to induce matrix deposition and TIMPs to prevent ECM deposition. It is thought that MSCs can reduce hypertrophic scarring by secreting HGF, FGF, adrenomedullin, and TGF-3 (Riedl et al., 2021). Stoff et al. (2009) found that after transplanting human MSCs into rabbit wounds, the wounds’ tensile strength rose and scar formation was greatly reduced. These results show that future MSC therapy may result in less scarring (Li et al., 2015).

## 8 The potential anti-infection effect and drug delivery system of MSCs in burn wound

Researchers are increasingly studying the potential anti-infection effect of MSCs in burn wounds, which can treat immune and inflammatory diseases caused by infection due to their paracrine function (Miao et al., 2021). Direct and indirect processes contribute to MSC antimicrobial activity. Direct methods involve the production of antimicrobial factors such as LL-37, and indirect methods involve the release of immunomodulatory substances that stimulate immune cell phagocytosis and the death of pathogens (Maxson et al., 2012).

Interestingly, MSCs are employed not only in regenerative medicine but also as carriers for drug delivery. MSCs offer advantages such as low immunogenicity, homing ability, and tumor tropism, making them ideal for targeted drug delivery systems (Matsuzaka and Yashiro, 2024). Despite the promising future of MSCs-DDS in targeting drug delivery, several inherent limitations of MSCs, such as poor drug loading capacity, limited homing efficiency, and potentials risk of living cells administration have so far restricted their practical applications for disease treatment (Senthilkumar et al., 2020).

Several novel technologies are being developed in parallel to improve the efficiency or safety of this system. Among technologies, nanotechnology and genome engineering are most employed to improve the drug loading capacity and homing efficiency of MSCs. Biomimetic technology has recently been proposed as a revolutionary approach to further improve the cell-based DDS, offering the superior benefits of high drug loading efficiency with similar or even better homing capability compared to cellular carriers, and also avoiding the potential risks of using living cells. Thus far, numerous technologies have been applied to overcome the aforementioned challenges of MSCs-DDS, and some of them have successfully improved the performance of MSCs in drug delivery (Su et al., 2021).

## 9 The immunomodulatory properties of MSCs

**Figure 2:**
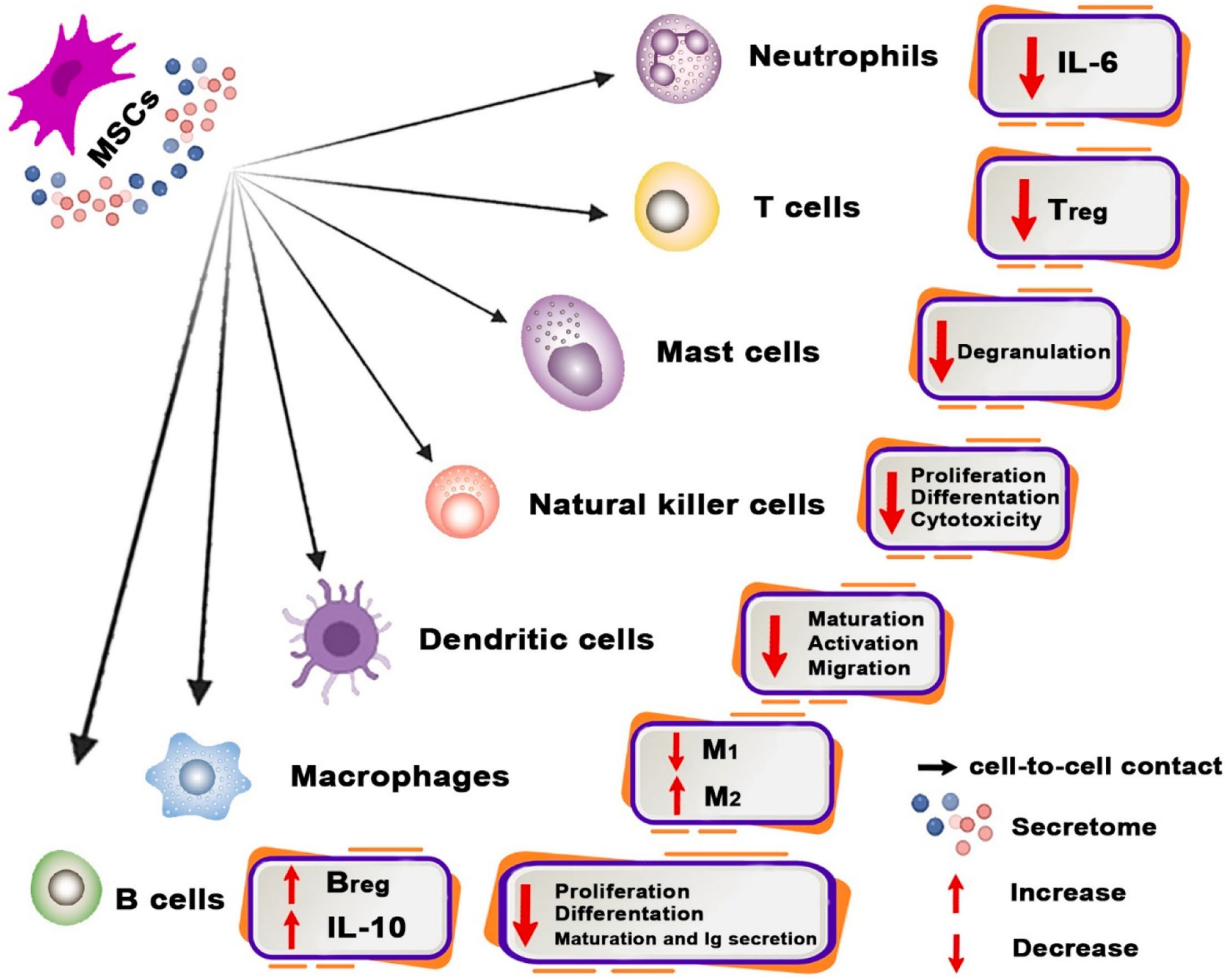
Immunomodulatory properties of MSC and related mechanisms. Immunomodulatory properties of MSCs possess the capability to modulate the functions of diverse immune system cells. MSCs facilitate the transition of macrophages from the pro-inflammatory M1 phenotype to the pro-healing M2 phenotype and regulate the development of dendritic cells (DCs) into a tolerogenic phenotype. Moreover, MSCs inhibit mast cell granulation and diminish IL-6 generation by neutrophils, as well as the proliferation, differentiation, and cytotoxicity of NK cells. Moreover, MSCs alter the phenotypic of B cells toward Breg and reduce Treg numbers.

One of the main characteristics of MSCs that makes them a desirable instrument for cell therapy is their immunomodulatory capabilities (Sarsenova et al., 2022). In 2002, Bartholomew et al. conducted the first study to demonstrate that MSCs reduce lymphocyte proliferation *in vitro* and increase skin graft survival *in vivo* (Radmanesh et al., 2020). MSCs have the ability to sense an injury and activate both innate and adaptive immunity, even if the response is weak. In addition, if the immune cells in the wounded area are overactive, they can be suppressed. This function is also referred to as the “sensor and switcher of the immune system,” which is controlled by many mechanisms (Sarsenova et al., 2022) (Figure 2).

MSCs generally perform as immunomodulators by preventing pro-inflammatory or effector immune cells from proliferating and maturing and also by directing certain immune cells into tolerogenic and anti-inflammatory phenotypes (Sinha et al., 2023). MSCs demonstrate their ability to modulate the immune system by inducing functional alterations in many immune cell types, including macrophages, dendritic cells (DCs), neutrophils, mast cells, natural killer (NK) cells, T cells, and B cells (Díaz-Prado et al., 2011; Subhan et al., 2021).

### 9.1 Association of MSCs with innate immune cells

#### 9.1.1 Macrophages

Macrophages are a vital component of the innate immune response and play a major role in the control of the inflammatory response (Maranda et al., 2017). Macrophages may be categorized into pro-inflammatory M1 and anti-inflammatory M2 macrophages based on their phenotypic and functional characteristics (Mirshekar et al., 2023). In contrast to M1 macrophages, which secrete pro-inflammatory cytokines such as IL-6, IFN-*γ*, TNF-*α*, and iNOS, M2 macrophages suppress inflammatory responses and hasten the healing of wounds by secreting a plethora of anti-inflammatory cytokines, including IL-4, IL-10, and arginase-1 (Arg-1) (Maranda et al., 2017).

Multiple studies have shown that MSCs have the ability to diminish the production of pro-inflammatory cytokines in macrophages, hence inducing a shift in macrophage polarization toward the anti-inflammatory M2 phenotype both *in vivo* and *in vitro* (Tamama and Kerpedjieva, 2012). Many molecules, such as PGE2, indolamin-2,3-dioxygenase (IDO), IL-6, HGF, IL-1 receptor antagonist (IL-1RA), TSG-6, and TGF-*β*, contribute to effective M2 macrophage polarization promotion (Mirshekar et al., 2023). Toll-like receptors (TLRs) allow MSCs to sense various danger signals. MSCs react to excessive pro-inflammatory signals through TNF-α, IFN-γ, and IL-1β receptors. Thus, MSCs release cytokines that either stimulate or inhibit immune responses to preserve the immunological balance. TLR2, TLR3, TLR4, TLR7, and TLR9 are all expressed by MSCs. Depending on the tissue from which these TLRs originate, their expression levels differ considerably. MSCs’ pro-inflammatory or anti-inflammatory phenotype is TLR-type dependent. As an example, TLR4 activation results in a pro-inflammatory phenotype, whereas TLR3 activation results in an anti-inflammatory phenotype (Jiang and Xu, 2020).

#### 9.1.2 DCs

DCs are the most crucial antigen-presenting cells (APCs) (Yang et al., 2021). There are typically two phases for DCs: the immature phase and the mature phase. Phenotypic and functional changes distinguish these phases. When immature DCs that process antigens develop into mature DCs that present antigens, the adaptive immune system is activated. Mature DCs can release cytokines that boost the immune system and cause inflammation. Whereas immature DCs stop adaptive immune cells and help maintain immunological tolerance. Thus, DCs immunomodulation can be accomplished by controlling their migration and maturation (Alahdal et al., 2020; Liu X. et al., 2023). MSCs not only hinder monocyte differentiation into DCs, but they can also impede DC maturation, attraction, and migration (Alvites et al., 2022). It is known that MSCs impede this differentiation process by secreting PGE2 (Seo and Jung, 2016). MSCs limit DCs’ ability to process and present antigens by stopping mitogen-activated protein kinases *in vivo*. In addition, in a model of mice, MSCs increased Tregs and activated functional tolerogenic DCs (Peng et al., 2023).

#### 9.1.3 Neutrophils

MSCs have the potential to extend the activity and survival of neutrophils and enhance the synthesis of factors including TGF-β, IFN-α, and granulocyte colony-stimulating factor. Their activation and invasion are mostly influenced by the production of IL-6 and other variables, including CXCL1, CXLC2, CXCL5, and CXCL8 (Alvites et al., 2022). To prevent neutrophil infiltration into the wound, MSCs secrete IL-10. In addition, to prevent neutrophil rolling and transendothelial migration, MSCs release TSG-6, which binds to protein ligands (Seo and Jung, 2016).

#### 9.1.4 Mast cells

Research has shown that mast cells (MC) have close ties to scar formation and wound healing. At the start of an infection, activated MCs release certain particles, such as histamine, 5-hydroxytryptamine, heparin, trypsin, chymotrypsin, and others. They also attract other inflammatory cells, such as monocytes, macrophages, and neutrophils. Another crucial function of MCs is to identify and present foreign antigens to T and B lymphocytes. Thus, MCs play a role in adaptive immunity (Zhao et al., 2023).

#### 9.1.5 NK cells

NKs target HLA-negative cells and have the ability to lyse MSCs because their actions are dependent on signals sent by receptors linked to HLA molecules (Alvites et al., 2022). Notably, MSCs perform through IDO, PGE2, and TGF-β1 to hinder NK cell proliferation and function (Seo and Jung, 2016; Weiss and Dahlke, 2019).

### 9.2 8Association of MSCs with adaptive immune cells

#### 9.2.1 T cells

MSCs have the ability to promote the differentiation of T cells from a pro-inflammatory state to an anti-inflammatory one, primarily via the inhibition of lymphocyte proliferation and the generation of pro-inflammatory cytokines (Yang et al., 2021). MSCs impede the proliferation of activated helper T (Th) cells, which results in a reduction in the secretion of IFN-γ and IL-17 by Th1 and Th17 cells. In addition, increased IL-4 secretion by Th cells demonstrates that Th cells change from a pro-inflammatory to an anti-inflammatory phenotype (Wang et al., 2021). In CD4^+^ T cells co-cultured with MSCs, the Notch1/forkhead box P3 (FOXP3) pathway was shown to be activated, which increased the number of CD4^+^ CD25 (high) FOXP3^+^ cells and mediated the induction of Tregs. While suppressing TGF-β and IL-10 at the same time inhibits Treg induction in co-cultured cells, it indicates that the two factors have a significant impact on the immune-tolerance system (Yang G. et al., 2023).

#### 9.2.2 B cells

There are three subtypes of B cells: B1, B2, and regulatory B cells (Bregs). The majority of B1 cells develop in the fetal liver and include B1a and B1b subsets. B2 cells can be further divided into follicular B (FOB) and marginal zone B (MZB) cells, which originate from bone marrow (Wang et al., 2020). FOB cells develop into plasma cells, which then secrete antimicrobial antibodies with high affinity. B1 and MZB cells have the ability to synthesize natural antibodies either in a T-cell-dependent or non-T-cell-dependent way. Bregs is responsible for suppressing the immune system through the secretion of cytokines such as IL-10, IL-35, and TGF-β or the expression of molecules that act as negative stimuli, such as FasL and PD-L1 (Liu P. et al., 2023). The ability of Bregs to suppress Th1 and Th17 responses and induce FOXP3^+^ has been proven in earlier research (Liu et al., 2020). MSCs can control B-cell proliferation and differentiation and prevent B-cell apoptosis. The first evidence that they can directly interact with B cells to stop their proliferation and apoptosis was found by Anna Corcione et al. in 2006 (Wang et al., 2020).

## 10 Experimental studies and clinical practice

The effectiveness and safety of MSCs in treating burn wounds are being investigated in clinical studies. In 2005, Rasulov et al. performed the first human study on a middle-aged female patient. She had 40% TBSA burns, 30% of which were full-thickness burns. Deep tissue was injected with BM-MSCs that resembled allogenic fibroblasts. They observed a marked improvement in hemostasis and wound bed epithelization without serious side effects (Schulman et al., 2022; Ahmadi et al., 2019). The use of autologous BM-MSCs for the treatment of severe radiation burns has been reported by Lataillade et al. (2007). This group saw the expected clinical progression and no recurrence of inflammatory radiation in 2010 after performing five local MSC transplants in conjunction with skin autograft (Bey et al., 2010).

Similarly, Mancilla et al. (2015) used a fibrin spray containing MSCs derived from bone marrow to treat a young man with 60% total burns (Ahmadi et al., 2019; Schulman et al., 2018). In addition, Portas et al. (2016) validated the use of cadaveric BM-MSCs in treating the chronic radiation-induced skin lesion (Golchin et al., 2019).

Abo-Elkheir et al. compared the wound healing effects of BM-MSCs, UC-MSCs, and early excision and transplant in patients with full-thickness burns in a study. They demonstrated that using BM-MSC and UC-MSC therapies significantly improved the rate of healing in a patient with a thermal full-thickness burn as compared with conventional methods (Abo-Elkheir et al., 2017).

Brennan et al. (2017) showed that, in contrast to BM-MSCs, AD-MSCs exhibit greater angiogenesis and inferior osteogenic capabilities *in vivo*.

## 11 Novel technologies in MSC application methods

The major treatments for full-thickness skin defects are autologous skin grafting and flap grafting. Autologous skin grafting needs a large amount of skin and may be limited by significant skin defects or pathological skin disorders (Zhao et al., 2024). MSCs, when used in conjunction with other tissue engineering methods, can improve their effectiveness in repairing skin tissue. These methods include the following:

1. Scaffolds combined with MSCs produce remarkable results. Stem cells can get nutrients and exchange gases in a controlled environment provided by tissue scaffolds. Scaffolding biomaterials must be tailored to the specific needs of the target tissue (Katiyar et al., 2022; Wang et al., 2021).

For example, chitosan and collagen together can create a scaffold that can even cure wounds completely, overcoming the drawbacks of conventional collagen scaffolds (Wu et al., 2024). Daniela et al. (2014) described mesenchymal stem cells (MSCs) seeded onto nanofibers as a potential novel alternative to split-thickness autologous skin grafting. This research demonstrated that the scaffolds increased cicatrization and extended MSC function (Steffens et al., 2014). Gholipour-Kanani (2012, 2014) and Shokrgozar (2012) introduced porous scaffolds populated with xenogeneic human and allogeneic mesenchymal stem cells, respectively. They assessed a quicker wound healing rate and histologically improved re-epithelialization compared to the scaffold-only group. Nonetheless, these investigations did not use any statistical analysis (Rangatchew et al., 2021). Clover et al. (2015) demonstrated that BM-MSC-seeded scaffolds markedly enhanced the wound healing rate by day 14 post-transplantation.

1. Compared to monolayer culture, MSCs are cultured in three-dimensional (3D) bioprinting scaffolds, which increase the synthesis of anti-inflammatory compounds (Riedl et al., 2021). Furthermore, research has demonstrated that 3D living dressings can control the immune response and stimulate neovascularization, two features essential to successful treatment (Turner et al., 2022). Materials such as polycaprolactone (mPCL) and polyethylene terephthalate (PET) can be used to 3D bioprinting scaffolds that efficiently transfer stem cells to the intended healing location (Brennan et al., 2017).
2. Hydrogels are water-absorbing polymers that maintain a moist environment, which is crucial for burn wound healing. Their properties include the following: (1) Moisture retention prevents desiccation and promotes cell migration. (2) Thermal regulation provides a cooling effect and protects against temperature fluctuations. (3) Biocompatibility reduces irritation and supports cellular interactions (Olteanu et al., 2024). (4) Hydrogels also have the potential to serve as the primary raw material for delivery carriers due to their skin-like rheological characteristics (Chai et al., 2017). Hydrogel dressings serve as carriers for mesenchymal stem cells (MSCs), facilitating their localized and sustained release. Research has demonstrated that this combination speeds up the healing process and decreases the amount of time it takes for epithelialization to occur. The anti-inflammatory properties of MSCs combined with the moist environment of hydrogels result in improved cosmetic outcomes (Khayambashi et al., 2021; Li Q. et al., 2022). Ozpur et al. (2016) used fibrin hydrogel containing keratinocytes and AD-MSCs to create *in vitro* skin tissue in one study. The results showed that the dermal substitute with AD-MSCs improved blood vessel growth and covered the wound again (Ozpur et al., 2016).
3. Modifying MSCs genetically can enhance their immunomodulatory activities and stimulate skin regeneration. It is possible to alter specific pathways, overexpress or suppress gene expression, or both in MSCs through genetic modification. Researchers have demonstrated that modifying MSCs using CRISPR/Cas-based non-viral gene editing enhances the transplanted MSCs’ survivability by elevating HIF1a gene levels (Riedl et al., 2021). Overcoming restrictions in editing efficiency and cytotoxicity, the CRISPR-Cas9 method enables accurate genome editing in MSCs. The development of genetically engineered MSCs with enhanced therapeutic capabilities has been made possible by ribonucleoprotein (RNP) delivery techniques, which have demonstrated low cell death and high indel frequencies (Han et al., 2024). MSCs can be engineered to overexpress growth factors, including PDGF-B, to improve their capacity to aid in wound healing (Kosaric et al., 2017).

## 12 The challenges of using MSCs on burn wounds

The treatment of MScs is not without challenges:

1. The issue pertains to both mass production and accessibility. Cell therapy methods typically use MSCs for tissue regeneration, and each treatment typically requires hundreds of millions of MSCs. As a result, larger bioreactors and longer *in vitro* cell culture growth are required (Miatmoko et al., 2023).
2. The immune system of the receiver may be impacted by the use of stem cells. The immunogenicity of the cells may change when given in non-physiological places (Miatmoko et al., 2023).
3. MSCs from diverse donors vary in proliferation, immunomodulation, and secretion. Thus, using MSCs from several unrelated donors makes it impossible to standardize and compare clinical trial data. A homogeneous cell supply with steady phenotypic and functional properties would be beneficial for regular MSC-based cellular treatment with consistent effects (Teshima, 2024).
4. Another danger is the transmission of bacterial, viral, fungal, or prion infections from the donor to the receiver, which can be threatening and even fatal (Miatmoko et al., 2023).
5. A number of issues limit the usefulness of *in vivo* models, including the high expense, difficulties in controlling and standardizing experimental conditions, and ethical issues surrounding the use of animals. Moreover, disparities between animal and human physiology may influence the applicability of findings to clinical settings. It is essential to weigh the benefits and drawbacks of *in vivo* models to obtain significant and pertinent insights into the mechanisms of burn wound healing (Das et al., 2024). Ultimately, researchers must explore alternative methodologies, such as *in vitro* models or computational simulations, that may provide more controlled environments and reduce ethical concerns. By integrating these approaches, the scientific community can enhance the reliability and relevance of their findings, leading to improved strategies for managing burn injuries in humans.
6. There are various ethical issues with the use of MSCs, particularly those obtained from allogeneic sources such amniotic fluid and umbilical cord:

- The procurement of MSCs from the umbilical cord and amniotic fluid often transpires during parturition. It is critical to make sure that parents are aware of the hazards and benefits of cell research before they give their consent (Corsano et al., 2015).
- As stem cell therapies become more commercialized, growing financial incentives for biological material donation raise concerns about potential exploitation of donors. This prompts questions about the commercialization of human tissues and the validity of consent. Therapies produced from these MSCs may not be accessible to everyone. Advanced treatments may be more beneficial to wealthier people or communities, which could exacerbate already-existing health disparities (Corsano et al., 2015; Mitrossili et al., 2015).

Due to the serious moral and legal concerns raised by MSCs derived from allogeneic origins, a worldwide regulatory framework is urgently required. We must promote equal access to these precious resources while also ensuring respect for individual dignity and autonomy (Mitrossili et al., 2015).

## 13 Conclusion

MSCs possess considerable potential in improving the repair of burn injuries owing to their regenerative capabilities. Large-scale clinical trials often lack control groups, which complicates conclusive determinations regarding efficacy and safety. The heterogeneity in MSC qualities due to donor features and isolation methods can result in variable therapeutic effects. In the future, researchers should work on standardizing how to treat MSCs and looking into combination therapies that use MSCs along with other ways to repair cells, such as biomaterials or gene therapy. By addressing these issues, researchers can enhance the reliability of MSC therapies and potentially improve patient outcomes. This approach may lead to more effective treatment options for burn injuries and other regenerative applications.
